# Calorie restriction increases telomerase activity, enhances autophagy, and improves diastolic dysfunction in diabetic rat hearts

**DOI:** 10.1007/s11010-015-2327-0

**Published:** 2015-02-07

**Authors:** Naoki Makino, Jun-ichi Oyama, Toyoki Maeda, Masamichi Koyanagi, Yoshihiro Higuchi, Keiko Tsuchida

**Affiliations:** Division of Molecular and Clinical Gerontology, Department of Molecular and Cellular Biology, Medical Institute of Bioregulation, Kyushu University, 4546 Tsurumihara, Beppu, 874-0838 Japan

**Keywords:** Calorie restriction, Diabetes, Autophagy, Diastolic function

## Abstract

The aims of this study were to investigate the impact of caloric restriction (CR) on cardiac telomere biology in an animal model of diabetes and to examine the signal transduction involved in cell senescence as well as cardiac function. Male 8-week-old Otsuka Long-Evans Tokushima fatty (OLETF) diabetic rats were divided into two groups: a group fed ad libitum (OLETF-AL) and a group fed with CR (OLETF-CR: 30 % energy reduction). Long-Evans Tokushima Otsuka (LETO) non-diabetic rats were used as controls. LETO rats were also divided into two groups: a CR (LETO-CR) group and a group fed AL (LETO-AL). At 40 weeks of age, the body weight was decreased by 9.7 % and the insulin resistance was less in OLETF-CR rats. Telomerase activity in OLETF-CR rats was significantly increased, and telomerase reverse transcriptase was more highly expressed in those rats. However, the telomere length (TL) was not different between AL- and CR-treated rats of each strain. The protein expressions for FoxO1 and FoxO3 were increased in OLETF-AL rats, but the levels of phosphorylated (p)-Akt were decreased compared to those in OLETF-CR rats. Autophagic LC3II signals revealed significant increases in OLETF-CR rats. Echocardiography showed that OLETF-CR improved the left ventricular diastolic dysfunction without changes in the left ventricular dimension. This study revealed that CR increases cardiac telomerase activity without TL attrition, and significantly ameliorates diastolic dysfunction. These findings suggest that cardiac telomerase activity may play an important role in the maintenance of normal cardiac function.

## Introduction

In a previous study using a rat model of type II diabetes mellitus (DM) rats model, diabetic cardiomyopathy was characterized functionally by the presence of left ventricular (LV) diastolic dysfunction, and histologically by interstitial fibrosis and collagen accumulation [1]. Although coronary artery diseases are the main cause of heart failure and deteriorating function, the high incidence of diabetic cardiomyopathy indicates that diabetes itself is an important factor in myocardial damage. Conventional therapeutic practices, such as strict control of blood glucose level and avoidance of traditional risk factors, are often effective, but not completely prevent cardiac complications [2]. Calorie restriction is commonly recommended for prevention and amelioration of diabetes. Caloric restriction (CR) has various beneficial effects on health, including lifespan prolongation [3]. One possible mechanism of the beneficial effects of CR is attenuation of mitochondrial dysfunction under various pathological conditions [4]. Several studies conducted on laboratory rodents have shown that CR promotes longevity and ameliorates the age-associated impairment of LV diastolic function, arterial elasticity and heart rate variability [5, 6].

In recent years, the role of telomere length (TL) in the pathogenesis of cardiovascular disease and diabetes as attracted increasing research interest. Telomeres, the tandem repeats of the TTAGGG DNA sequence extending at the end of the eukaryotic chromosomes, undergo attrition during every cell division and their length is the best indicator of the replication potential of somatic cells [7–9]. The present work offers a proof of concept supporting the validity of the model that the difference between muscle TL and TL of proliferative tissues (that is leukocytes or skin) provides information over and above that of cross-sectional analysis of age-dependent TL shortening in proliferating tissues.

While the mammalian heart has low, but functionally significant, levels of telomerase expression, the cellular population responsible remains incompletely characterized [10]. Emerging evidence also indicates that telomerase activity plays an important role in the development and function of the normal heart; *TERC* ^−/−^ mice that lack telomerase activity develops cardiac abnormalities, including dilated cardiomyopathy and reduced angiogenic potential [11]. We previously observed that cardiac telomerase activity was associated with the insulin resistance in an experimental rat model of diabetes [12].

Aging is known to increase the prevalence of metabolic disorders such as diabetes [8]. Cellular aging is considered to influence insulin resistance and this can lead to excessive calorie intake, obesity, and diabetes. It is now apparent that cellular senescence can be induced by various stressors that are independent of cell replication, such as chromatin damage related to oxidative stress. Furthermore, accumulating evidence also suggests a potential relationship between cellular senescence and the aging of organisms [13]. Autophagy is also involved in various pathophysiological processes, and shows increased activity in response to extracellular and intracellular stimulations such as CR. Autophagy increases in response to acute and chronic myocardial ischemia, heart failure, and cardiomyopathic degeneration [14].

The effect of CR on telomerase in the heart has not previously been reported, and here we tested the hypotheses that long-term CR can affect telomere biology as well as diastolic function in diabetes using a rat model of spontaneous type II diabetes, Otsuka Long-Evans Tokushima fatty (OLETF) rats. OLETF rats develop hyperglycemic obesity with hyperinsulinemia and insulin resistance after the age of 25 weeks, similar to patients with non-insulin-dependent DM [1]. To gain a better understanding of the molecular mechanisms, we also examined whether CR could modify the expression of senescence-related molecules to ensure telomere integrity and thereby maintain tissue homeostasis and regulate lifespan.

## Methods

### Animal protocol

Experiments using animal subjects or tissues from animals were conducted in accordance with the *Guide for the Use and Care of Laboratory Animals* (NIH Pub. No. 85–23, Revised 1996) and were approved by the Institutional Animal Care and Use Committee of Kyushu University. OLETF rats, established genetic diabetic model of human type II [1], were used for all experiments in this study. Male OLETF rats at 5 weeks of age were supplied by the Tokushima Research Institute, Otsuka Pharmaceutical (Tokushima, Japan) and upon arrival were randomly assigned to sedentary cage conditions with ad libitum feeding for 3 weeks. At 8 weeks of age, OLETF rats were divided into two groups: one group was maintained for 32 weeks under cage conditions with ad libitum feeding (OLETF-AL), and the other group was maintained under the same conditions but with food intake reduced to 70 % of normal (OLETF-CR). The daily amount of food intake was assessed in the rats with ad libitum feeding and was adjusted in the CR rats according to increasing body weight to maintain a food intake of 70 % of ad libitum feeding. Non-hyperphagic, control-strain Long-Evans Tokushima Otsuka (LETO) rats were used as controls and were maintained under sedentary cage conditions. The LETO rats were also divided into two groups: a group with ad libitum feeding (LETO-AL), and a CR group with food intake reduced to 70 % of normal (LETO-CR). All rats had free access to standard laboratory chow (MF; Oriental Yeast, Tokyo, Japan) and tap water, and were taken care of under the specifications outlined in the Guiding Principles for the Care and Use of Laboratory Animals. All rats were caged individually in an environment in which temperature (23 ± 2 °C) and humidity (55 ± 5 %) were controlled and an artificial light cycle was used. During the study period, both body weight and systolic blood pressure were measured by the tail-cuff method (BP-98A; Softran, Tokyo, Japan) once a week. For the analysis of blood samples, blood was collected from the tail vein between 10:00 AM and noon under nonfasting conditions during the study. All serum samples were stored at −80 °C until analysis. Plasma glucose levels were measured using the glucose oxidase method. Plasma insulin levels and serum adiponectin levels were measured using an ELISA kit (Linco Research, St. Charles, MO). As a marker of insulin resistance, the homeostasis model assessment of insulin resistance (HOMA-IR), which was determined based on both plasma glucose and serum insulin levels, was analyzed. At the end of the study, the animals were killed by decapitation. The liver and heart tissues were then excised. All excised tissues were immediately frozen in liquid nitrogen and stored at −80 °C until used for analysis.

### Echocardiography

Each rat was lightly sedated with 3–4 % isoflurane, and then the chest was shaved and the rat was placed in the supine position. After anesthesia with intraperitoneal sodium pentobarbital (50 mg/kg), echocardiography was performed using an LOGIQ 400 PRO ultrasonography system (GE Medical Systems, Milwaukee, WI) equipped with a 10–12 MHz transducer, as previously described [12, 15]. The transmitral flow velocity profile was determined by positioning a sample volume at the tip of the mitral valve on the apical four chamber view. The Doppler beam was set within <15° of the incident angle to the flow direction identified on the color Doppler image. Angle correction was performed by a semiautomated system installed in the machine. The peak velocity (*E*), deceleration time (DT) of the early diastolic filling wave, and early mitral annulus velocity (*E*′) were measured [16, 17]. DT was obtained by extrapolating the initial slope of early diastolic filling wave deceleration to the baseline [1]. *E*′ was measured at the septal portion of the mitral annulus in an apical four chamber view, using a tissue Doppler technique with a Nyquist limit of 15 cm/s. The transmitral inflow pattern was recorded on a strip chart at 100 mm/s sweep speed with simultaneous 3-lead electrocardiography for offline analysis. All measurements represented the mean of 5 consecutive cardiac cycles, and heart rate was calculated on the basis of the strip chart of Doppler echocardiography.

### Genomic DNA extraction

Rat tissue samples were lysed by incubation at 55 °C for 48 h in 200 ul lysis buffer containing 10 mmol/l Tris HCl (pH 8.0), 0.1 mmol/l EDTA (pH 8.0), 2 % SDS, and 500 g/ml protease K (Roche Diagnostic, Tokyo, Japan). Genomic DNA extraction was performed using a DNeasy tissue kit (Qiagen, Tokyo, Japan), according to a previous publication [18].

### Telomere biology

TL was estimated as the telomeric-to-centromeric DNA content ratio in dot blot analysis, as previously reported [12]. The telomeric DNA content can be standardized by calculating the telomeric DNA content relative to the centromeric DNA (0.1 μg) content. DNA samples were diluted and denatured. 2 μl of denatured DNA was dotted onto a nylon membrane sheet. The hybridization signal of a DIG-labeled probe was converted into a chemiluminescent signal using a DIG Wash and Block buffer set and a DIG luminescent detection kit (Roche). The telomere probe was used as previously described [12]. Telomerase activity was examined using a modified telomerase repeat amplification protocol (TRAP) [12, 15] with TeloChaser (Toyobo, Osaka, Japan), according to the manufacturer’s instructions. Briefly, the substrate oligonucleotide is added to 0.5 g protein extract. If telomerase is present and active, telomeric repeats (GGTTAG) are added to the 3′-end of the oligonucleotide. After amplification, the PCR products were resolved on a 12 % polyacrylamide gel, stained with ethidium bromide and detected using an FLA 5000 system (Fuji Film, Tokyo, Japan). The intensities of the bands were quantified with Image J software. For each group of rats, telomerase activity was analyzed in 7 tissue types from 6 animals. Assays were repeated at least twice for each individual rat to ensure reproducibility. A human cancer cell line overexpressing telomerase was used as the reference in each assay.

### Western blot analyses

The Western blot analyses were carried out according to the methods described in our previous reports [12, 15]. Briefly, the tissues were homogenated with 5 volumes of homogenization buffer (RIPA), and the supernatants were fractionated by SDS-PAGE. The proteins were transferred to nitrocellulose membranes (162-0112; Bio-Rad Laboratories, Hercules, California), and blocked with 5 % dry milk or blocking solution for Western blot (Roche). The membranes were then exposed to rabbit polyclonal immunoglobulin G (IgG) TRF2 (H-300: sc-9143; Santa Cruz Biotechnology, Santa Cruz, CA; dilution 1:200), rabbit polyclonal IgG anti-p53 (FL-393: sc-6243;Santa Cruz Biotechnology; dilution 1:500 in dry milk 1 %), rabbit polyclonal IgG anti-phosphorylated (phospho)-Akt (Ser473) (#9271; Cell Signaling Technology, Danvers, MA; dilution 1:1,500), anti-Mn-SOD (1:10,000; catalog number SOD-111; StressGen, Victoria, Canada), anti- phospho-FoxO1 Ser-256 (1:700; catalog no. 9461; Cell Signaling), anti-light chain (LC) 3 (1:1,000; catalog no. 9215; Cell Signaling), anti–cleaved caspase 3 (Cell Signaling Technology), or anti-Beclin1 and mouse monoclonal IgG glyceraldehyde-3-phosphate dehydrogenase (GAPDH) (6C5: sc-32233; Santa Cruz Biotechnology; dilution 1:1,000). For analysis of reverse transcriptase catalytic subunit (TERT) and an associated RNA component (TERC), immunoprecipitations were performed using polyclonal IgG anti-TERT or anti-TERC (H-231: sc-7212; Santa Cruz Biotechnology) and agarose-A protein goat anti-rabbit IgG (Sigma-Aldrich). Immunodetection was performed using goat anti-rabbit IgG (Sigma-Aldrich) and goat anti-mouse (170-6516; Bio-Rad) secondary antibodies (1:4,000 dilution), and an enhanced chemiluminescence kit (Amersham Biosciences).

### Immunohistochemistry

For histological analysis, heart tissues (*n* = 6 in each group) were immersed in 10 % buffered formalin. The fixed tissues were dehydrated, embedded in paraffin, sectioned into 4 μm slices and stained with Masson’s trichrome stain. To detect DNA damage in rat hearts subjected to CR, an antibody to 8-hydroxydeoxyguanosine (8-OHdG) (Oxis International) was used according to a previously reported procedure [4, 15]. To evaluate autophagy, immunofluorescence staining for LC3 was detected in heart tissue using a primary antibody to rat LC3 (No. 010-22841, 1:400) [15]. Anti-rabbit IgG (Molecular Probes AlexaFluoro488) was used for the secondary antibody. Nuclei were stained with 4′,6-diamino-2-phenylindole (DAPI) where indicated. Fluorescence imaging was performed with an Olympus BX51 (Olympus) equipped for epi-fluorescence microscopy with a CCD camera. The 8-OH-dG or LC3 signals were quantified, and the data expressed as particle number/area fraction [16].

### Statistical analysis

Data are expressed as the mean ± SEM. Statistical comparisons among the experimental groups were performed using a Student’s *t* test or one-way analysis of variance. Values of *p* < 0.05 were considered significant.

## Results

The general characteristics of the animals used in the present study are shown in Table 1. The body weights of 8-week-old LETO rats were not significantly different between the AL and CR groups and were also not significantly different in the same aged OLETF rats between these treatment groups. At 40 weeks of age, the body weight was more increased in the OLETF-AL than the LETO-AL rats, and was significantly lower in both strains of rats fed a CR diet than an AL diet. The heart-to-body weight ratios were not significantly different between the OLETF-AL and OLETF-CR groups at 40 weeks of age, although those were significantly reduced in LETO-CR rats, compared with LETO-AL rats. Systolic blood pressure was significantly lower in OLETF-CR than OLETF-AL rats, but was not significantly different between 8-week-old rats of either strain and 40-week-old LETO rats. Fasting glucose levels were significantly greater in 8-week-old OLETF rats than LETO rats. At 40 weeks of age, these levels were significantly lower in OLETF-CR rats than OLETF-AL rats. The serum levels of insulin, HOMA-IR, total cholesterol, and triglyceride were significantly lower in OLETF-CR than OLETF-AL rats. However, the concentration of serum adiponectin was higher in the two strains of rats fed a CR diet than in those fed an AL diet at 40 weeks of age. The increase in adiponectin was greater in OLETF-CR than LETO-CR rats.

**Table 1:** General Characteristics in LETO and OLETF Rats fed AD or CR at 8 and 40 weeks of age

	LETO (8 weeks)		OLETF (8 weeks)		LETO (40 weeks)		OLETF (40 weeks)	
	AL (*n* = 6)	CR (*n* = 6)	AL (*n* = 6)	CR (*n* = 6)	AL (*n* = 6)	CR (*n* = 6)	AL (*n* = 6)	CR (*n* = 6)
Body Wt. (g)	245 ± 12	239 ± 11	246 ± 11	249 ± 16	445 ± 2	395 ± 25^a^	608 ± 31^a^	549 ± 29^ab^
sBP (mmHg)	126 ± 7.1	124 ± 5.4	121 ± 5.6	124 ± 5.8	130 ± 6.5	129 ± 5.6	148 ± 7.1^a^	134 ± 5.1^b^
Heart wt. (g)	–	–	–	–	1.24 ± 0.06	1.30 ± 0.07	1.41 ± 0.07	1.32 ± 0.08
H/B wt. ratio	–	–	–	–	2.39 ± 0.07	2.11 ± 0.10^b^	2.41 ± 0.10	2.41 ± 0.18
Glucose	98 ± 4.8	111 ± 7.1	141 ± 8.2^a^	138 ± 6.9^a^	108 ± 6.8	116 ± 5.8	178 ± 7.3^a^	139 ± 7.1^b^
Serum Insulin	2.2 ± 0.1	2.4 ± 0.1	7.6 ± 0.32^a^	8.1 ± 0.38^a^	2.3 ± 0.1	2.0 ± 0.1^b^	10.3 ± 0.5^a^	4.6 ± 0.4^ab^
HOMA-IR	0.52 ± 0.03	0.66 ± 0.05	2.64 ± 0.11^a^	2.74 ± 0.18^a^	0.61 ± 0.04	0.57 ± 0.04	4.53 ± 0.23^a^	1.58 ± 0.06^ab^
Total Cholesterol	108 ± 6	110 ± 5	121 ± 9^a^	126 ± 7^a^	111 ± 7	106 ± 6	158 ± 1^a^	129 ± 6^b^
Triglyceride	41 ± 2	45 ± 3	42 ± 2	38 ± 3	164 ± 10^a^	128 ± 5^ab^	241 ± 15^a^	153 ± 9^ab^
Adiponecin	2.1 ± 0.1	2.2 ± 0.1	1.8 ± 0.1	1.9 ± 0.1	1.9 ± 0.1	2.7 ± 0.1^ab^	1.5 ± 0.1^a^	3.2 ± 0.2^ab^

Values are mean ± SE

*AL* ad libitum; *CR* calorie restriction; *sBP* systolic blood pressure; *H/B wt. ratio* heart to body weight ratio (mg/g); *HOMA-IR* homeostasis model of assessment—insulin resistance, glucose; *Total cholesterol and Triglyceride* mg/dl; *Insulin* μU/ml; *Adiponectin* μg/ml

^a^ *p* < 0.05 versus 8 weeks old same strain rats fed AL

^b^ *p* < 0.05 versus 40 weeks old same strain rats fed AL

An echocardiographic study was performed in the two strains of rats fed an AL diet or a CR diet at 8 and 40 weeks of age (Table 2). The LV end-diastolic dimension, LV end-systolic dimension, and fractional shortening were not significantly different among all groups of rats, indicating that there was no alteration of systolic function even by the treatment with CR in both strains. With regard to LV diastolic function in 40-week-old OLETF-AL rats, the *E*/A ratio was significantly higher, the deceleration time of the *E* wave was significantly longer, E′ increased significantly, and *E*/*E*′ was significantly decreased. These changes were not observed in LETO-AL or LETO-CR, and were comparable with those in OLETF-CR rats. Collectively, these findings show that diastolic function was preserved or even improved in the OLETF-CR rats.

**Table 2:** Results of doppler echocardiography in LETO and OLETF Rats with AL or CR at 8 and 40 weeks of age

	LETO (8 weeks)		OLETF (8 weeks)		LETO (40 weeks)		OLETF (40 weeks)	
	AL (*n* = 6)	CR (*n* = 6)	AL (*n* = 6)	CR (*n* = 6)	AL (*n* = 6)	CR (*n* = 6)	AL (*n* = 6)	CR (*n* = 6)
HR (beats/min)	388 ± 18	394 ± 19	402 ± 21	392 ± 23	441 ± 19	428 ± 20	435 ± 21	418 ± 18
LVEDD (mm)	6.3 ± 0.3	6.2 ± 0.5	6.9 ± 0.5	6.7 ± 0.4	8.1 ± 0.4	7.9 ± 0.4	8.3 ± 0.5	7.9 ± 0.4
LVESD (mm)	3.9 ± 0.3	3.8 ± 0.2	4.2 ± 0.2	4.0 ± 0.2	4.6 ± 0.3	4.5 ± 0.3	4.7 ± 0.2	4.6 ± 0.3
FS (%)	39 ± 1.8	38 ± 1.7	39 ± 2.1	40 ± 2.4	43 ± 2.9	43 ± 2.4	43 ± 2.1	42 ± 2.4
*E* (cm/sec)	103 ± 8.7	104 ± 6.8	105 ± 5.9	101 ± 6.2	106 ± 6.3	107 ± 5.9	98 ± 5.4^a^	101 ± 7.1^ab^
A (cm/sec)	41 ± 2.8	42 ± 2.7	43 ± 1.8	41 ± 2.2	42 ± 2.6	44 ± 2.8	46 ± 2.1^a^	44 ± 2.9^b^
*E*/A	2.5 ± 0.18	2.4 ± 0.17	2.4 ± 0.16	2.4 ± 0.16	2.5 ± 0.15	2.4 ± 0.15	2.1 ± 0.15^a^	2.3 ± 0.18^b^
*E*′ (cm/sec)	10.1 ± 0.6	9.8 ± 0.5	10.3 ± 0.7	9.7 ± 0.5	9.7 ± 0.4	9.8 ± 0.6	12.5 ± 0.7^a^	10.6 ± 0.5^ab^
*E*/*E*′ ratio	10.4 ± 0.71	10.6 ± 0.60	10.2 ± 0.55	10.4 ± 0.66	10.9 ± 0.53	10.9 ± 0.56	7.8 ± 0.42^a^	9.5 ± 0.38^ab^
DT (m sec)	34 ± 1.2	35 ± 1.7	33 ± 1.7	34 ± 1.4	40.0 ± 2.2	39.4 ± 2.5	47.8 ± 2.8^a^	42.2 ± 2.9^ab^

Values are mean ± SE

*HR* heart rate; *LVEDD* left ventricular end-diastolic dimension; *LVESD* left ventricular end-systolic dimension; *FS* fractional shortening, FS was determined by the following equation: FS = [(LVEDD−LVESD)/LVEDD] × 100 (%); *E/A ratio* peak velocity of early transmitral inflow (E)-to-peak velocity of late transmitral inflow (A) ratio; *E’* early mitral annulus velocity; *DTE* decelaration time of the E wave

^a^ *p* < 0.05 versus LETO rats fed AL

^b^ *p* < 0/05 versus OLETF rats fed AL

**Fig. 1:**
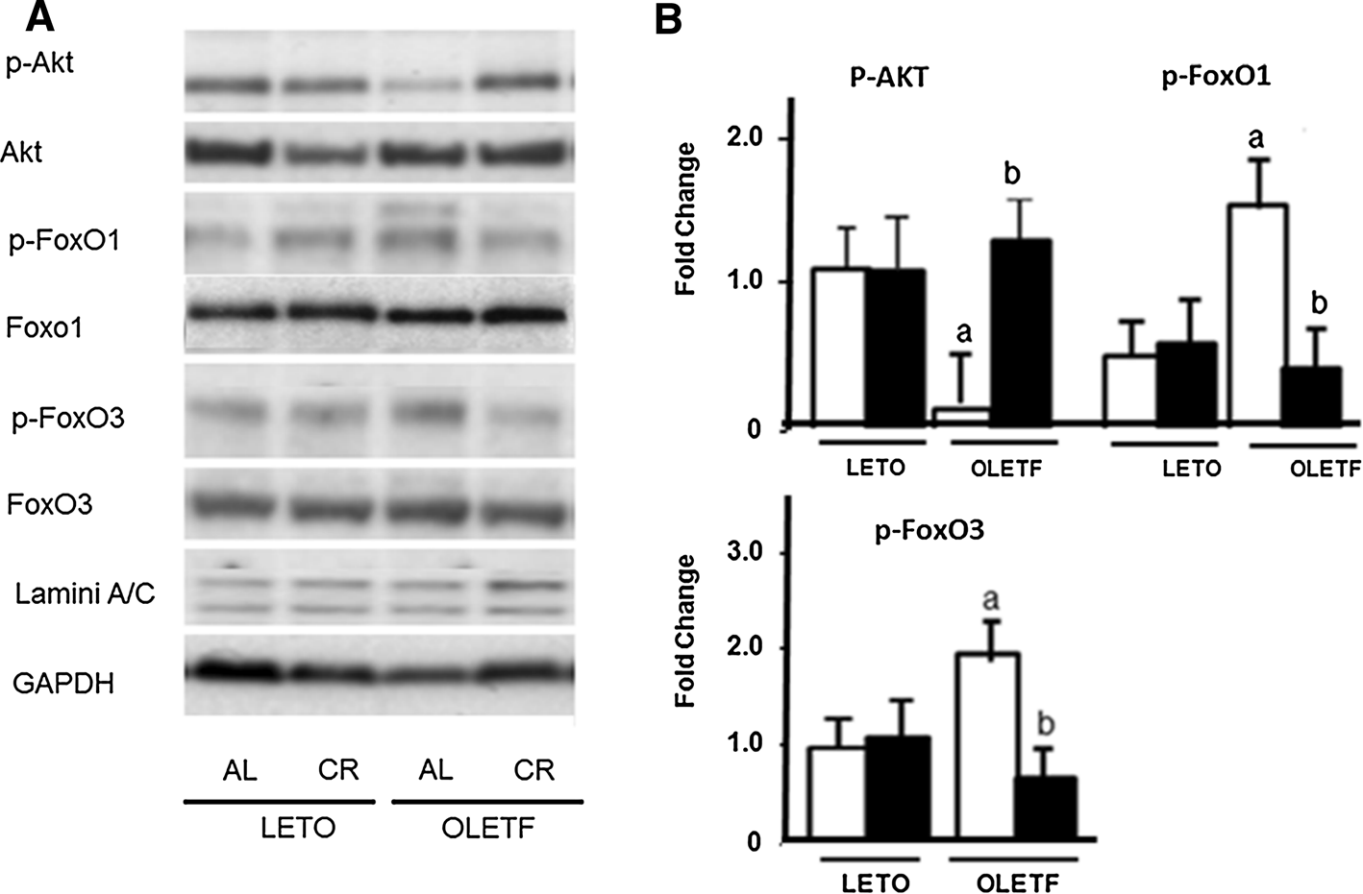
Protein expression of p-Akt, p-FoxO1, and p-FoxO3 in the hearts of LETO and OLETF rats with or without CR. **a** Representative results. **b** Summarized results. *Open bar* indicates rats fed ad libitum (AL) and *closed bar* indicates rats with calorie restriction (CR) in each strain. Each group contained 6 animals. Values are the mean ± SE. *a* *p* < 0.05 versus LETO-AL rats; *b* *p* < 0.05 versus OLETF-AL rats

**Fig. 2:**
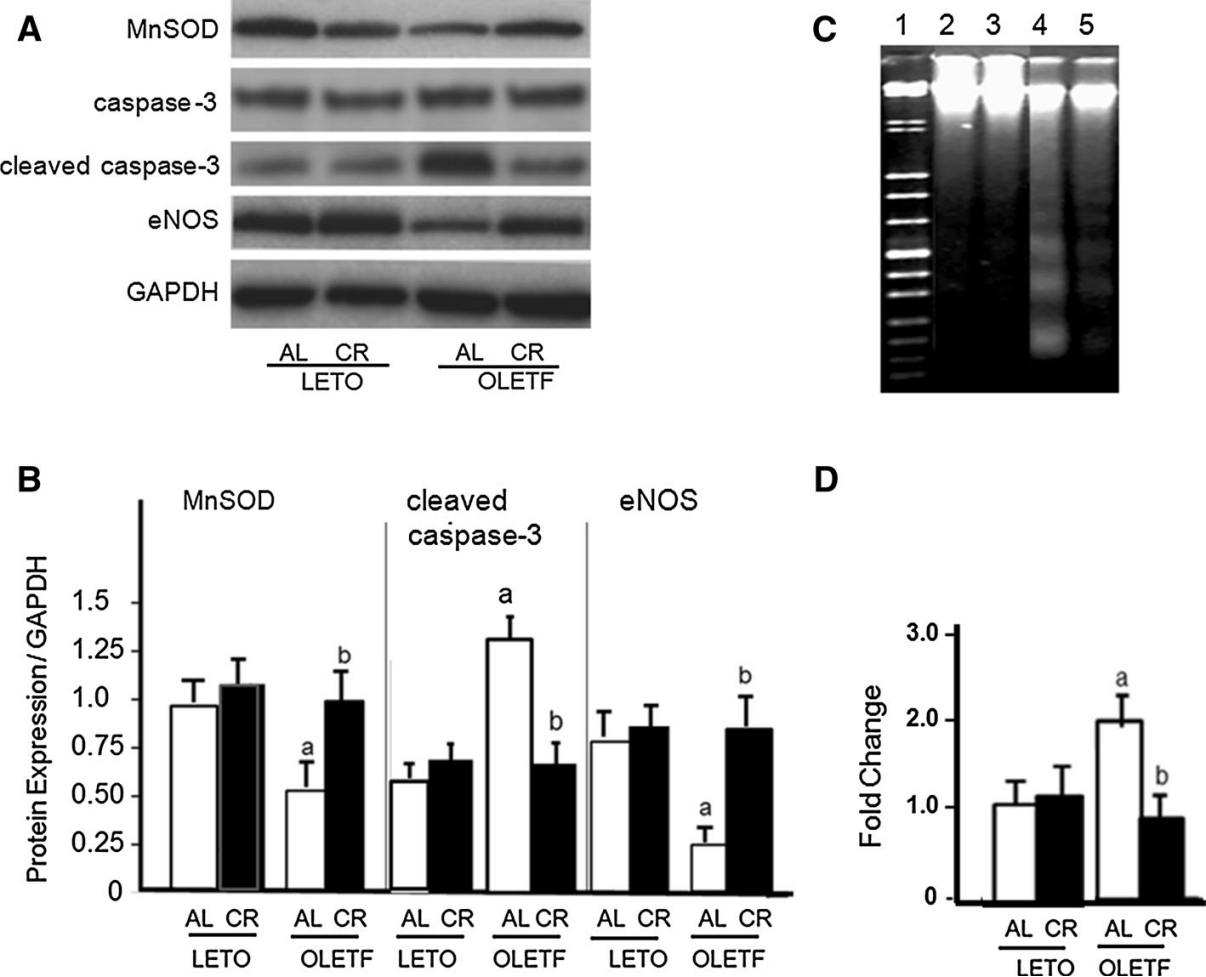
Protein expression of manganese superoxide dismutase (MnSOD), cleaved caspase -3, and endothelial nitric oxide (eNOS) in the hearts of LETO and OLETF rats with or without CR. **a** Representative results for Western blot analyses. **b** Summarized results in A. **c** The internucleosomic fragmentation in the hearts. *Lane 1* DNA marker. *Lane 2* LETO Al, *lane 3* LETO-CR, *lane 4* OLETF-AL, *lane 5* OLETF-CR. **d** Summarized results in **c**. Each *bar* indicates the same patterns presented in Fig. 1. Each group contains 6 animals. Values are the mean ± SE. *a* *p* < 0.05 versus LETO-AL rats; *b* *p* < 0.05 versus OLETF-AL rats

We also investigated the cell signaling for CR in the LETO and OLETF rats fed an AL or a CR diet (Fig. 1). The protein levels for p-Akt were lower in the OLETF-AL than in either the LETO-AL and LETO-CR rats. These reductions in expression were induced to enhance both of phospho-FoxO1 and phospho-FoxO3 in OLETF-AL rats, while those expressions were reduced in OLETF-CR rats. To further examine the antioxidant enzyme, manganese superoxide dismutase (MnSOD), cleaved caspase-3, indicators of cellular apoptosis, and endothelial nitric oxide synthase (eNOS) were also assessed by Western blot analysis (Fig. 2a). In OLETF-AL rats, the protein expressions of MnSOD and eNOS were significantly attenuated and those of cleaved caspase-3 were significantly enhanced. However, these changes were not observed in OLETF-CR rats (Fig. 2b). The DNA fragmentation in the hearts was also examined (Fig. 2c). The lane 4 from OLETF-AL rats indicated the enhanced fragmentations, but those of OLETF-CR (lane 5) did not (Fig. 2d). These results indicate that CR has beneficial effects for reduction of oxidant stress which was the increases of antioxidant enzymes and the inhibition of apoptosis in myocardium of OLETF rats.

**Fig. 3:**
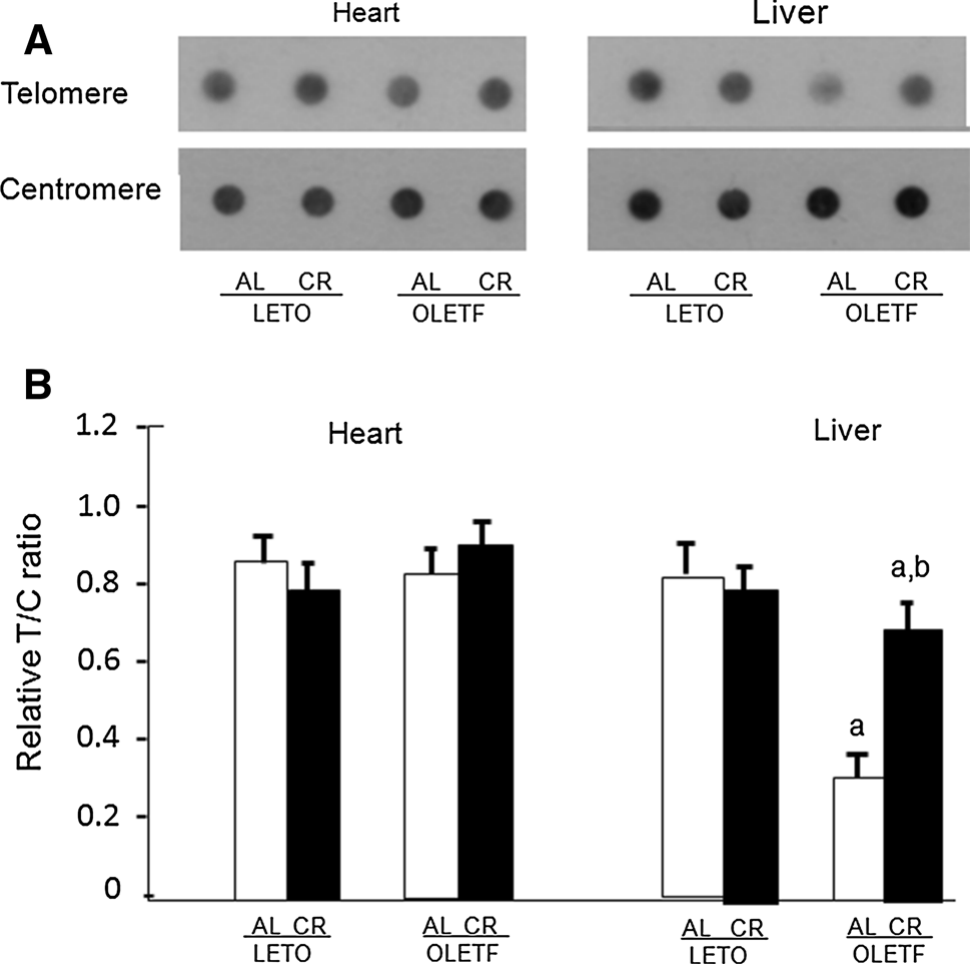
Length of telomeric DNA in heart and liver tissues from LETO and OLETF rats with or without CR. The length of telomeric DNA, as assessed by dot blot analysis, is presented as the telomeric-to-centromeric DNA content (T/C) ratio. **a** Representative data. **b** Relative T/C ratio. The *open bar* indicates data from LETO or OLETF rats fed ad libitum; the *solid bar* indicates data from LETO-CR or OLETF-CR rats. Each group contained 6 animals. Values are the mean ± SE. *a* *p* < 0.05 versus LETO-AL rats; *b* *p* < 0.05 versus OLETF-AL rats

**Fig. 4:**
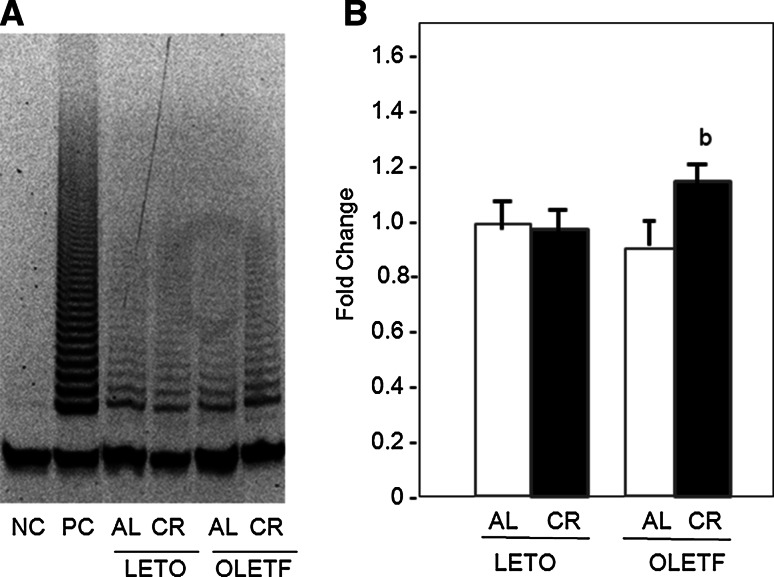
Telomerase activity in heart tissue from LETO and OLETF rats with or without CR. Telomerase activity was quantified using a telomerase repeat amplification protocol assay. **a** Representative results. **b** Summarized results obtained from 6 experimental data. The level for data of LETO-AL rats is taken as 1.0. *NC* negative control, *PC* positive control. Values are the mean ± SE; *b* *p* < 0.05 versus OLETF-AL rats

**Fig. 5:**
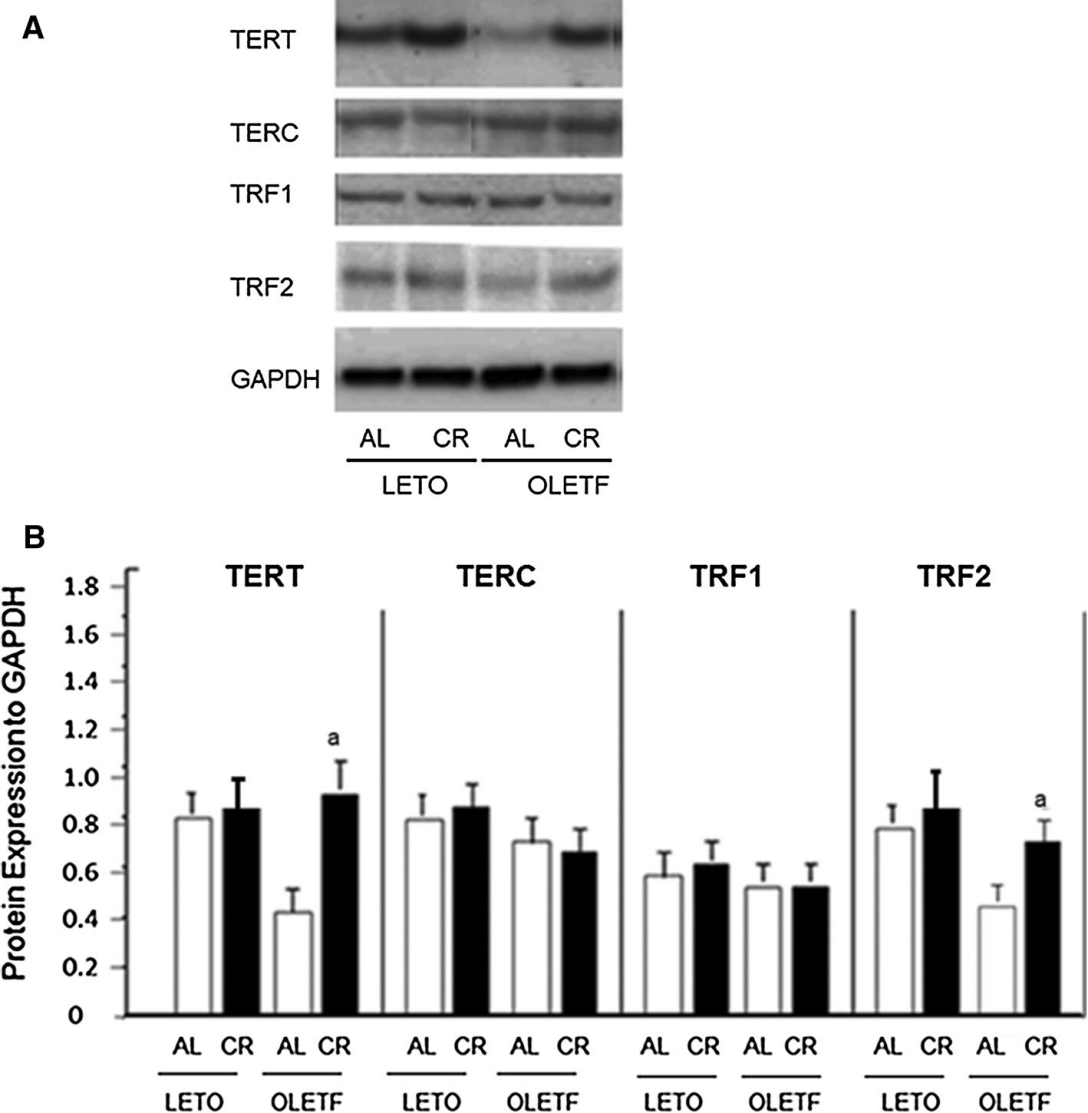
Protein expression levels of telomere reverse transcriptase (TERT), telomere RNA component (TERC), telomere repeat binding factor TRF1 and TRF2 in heart tissue from LETO and OLETF rats with or without CR. **a** and **b** representative (**a**) and summarized results presented as a ratio of protein expression to GAPDH (**b**). Values are the mean ± SE. *a* *p* < 0.05 versus LETO-AL rats; *b* *p* < 0.05 versus OLETF-AL rats

To evaluate the effects of OLETF-CR on telomere biology in rat models of diabetes, the TL was assessed by dot blot analysis of heart and liver tissues at 40 weeks of age (Fig. 3). The TL was significantly reduced only in the liver tissue of OLETF-AL rats, and was significantly increased by OLETF-CR. However, the TL attrition was not observed in heart tissue between OLETF-AL and OLETF-CR rats. There were no differences in the TL in either the heart or liver tissues between LETO-AL and LETO-CR. Next, telomerase activity, quantified using a TRAP assay, was found to be significantly elevated in the hearts of OLETF-CR rats compared to OLETF-AL rats, but this activity was not different between LETO-AL and LETO-CR rats (Fig. 4). To further evaluate telomerase activity in the heart tissue, the associated protein expression levels of the catalytic subunits TERT and TERC and associated proteins were examined (Fig. 5a). The expression levels of TERT and TRF2 were lower in OLETF-AL rats than LETO-AL rats. However, the reductions in TERT and TRF2 seen in OLETF-AL were inhibited in OLETF-CR rats. There were no significant differences in the expressions of TERC or TRF-1 among the groups (Fig. 5b).

**Fig. 6:**
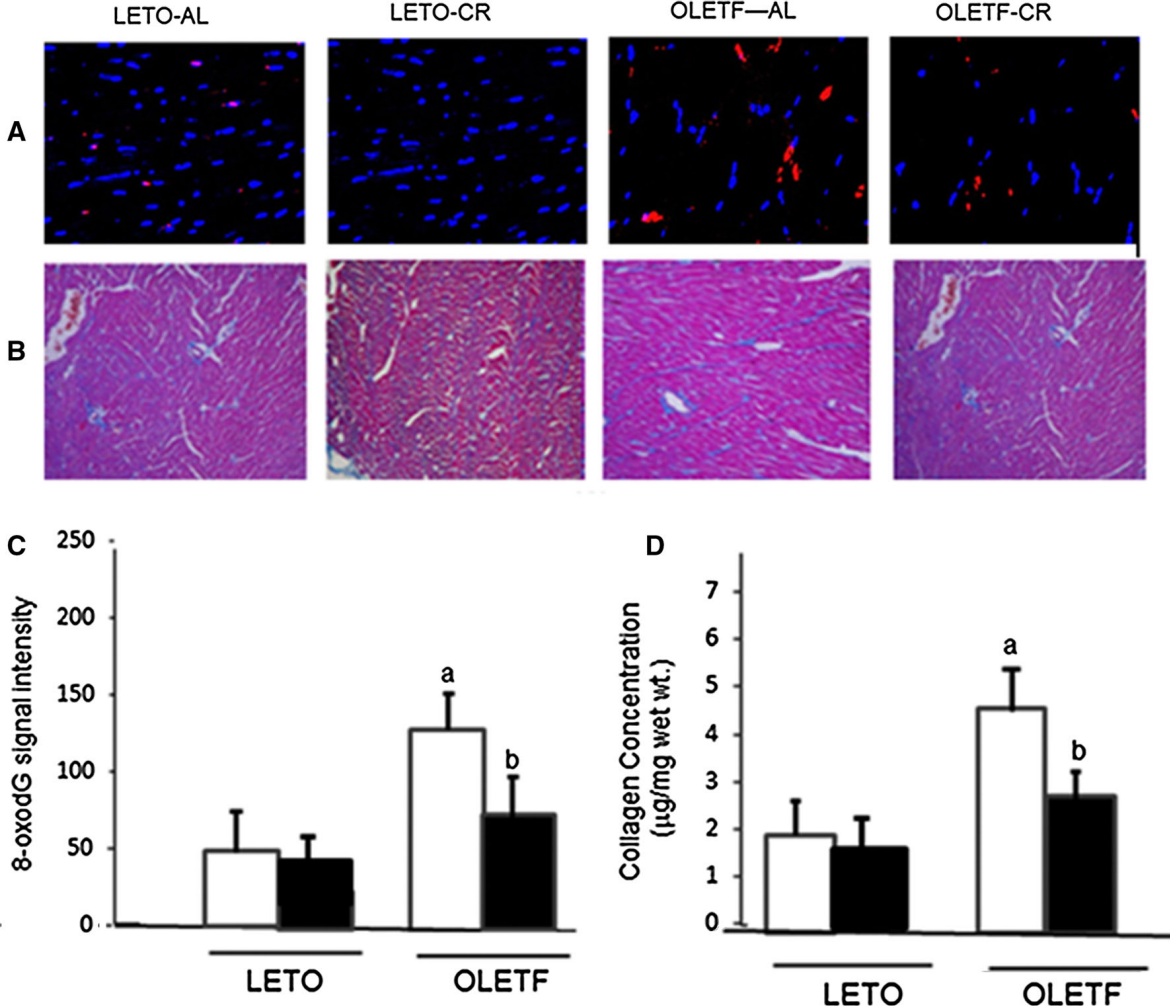
Histological findings in heart samples from LETO and OLETF rats with or without CR. Each picture shows LV cross-sectional views of 8-oxodG staining (*red*) as a marker of oxidant stress in nuclei (**a**). The positions of nuclei are determined by DAPI staining (*blue*). Original magnification, ×400. LV cross-sectional views of Masson’s trichrome staining (×100) (**b**). The summarized 8-oxodG signal intensities of DNA damage (**c**). Cardiac fibrosis in heart sections from experimental rats was determined as collagen concentration (g/mg dry wt) in the left ventricle of 5 animals (**d**). Values are the mean ± SE for 6 experiments. *a* *p* < 0.05 versus LETO-AL rats; *b* *p* < 0.05 versus OLETF-AL rats. (Color figure online)

To investigate whether CR affected oxidative DNA damage in the hearts of the diabetic animals, immunostaining with 8-OHdG, a biomarker of oxidative DNA damage, followed by DAPI staining, was performed (Fig. 6a, c). The signal intensities of nuclear DNA damage (red particles) were significantly lower in the heart tissue from OLETF-CR rats, compared with that of OLETF-AL rats. By the way, those signals were no difference between LETO-AL and LETO-CR. These results suggest that the oxidative damage in nuclei was attenuated by OLETF-CR. To quantify the extent of myocardial fibrosis in experimental rats, LV cross-sectional views were examined by Masson’s trichrome staining (Fig. 6b). Cardiac fibrosis in heart sections from experimental rats was also determined as collagen concentration (Fig. 6d). The collagen concentration was significantly higher in OLETF-AL rats than in LETO rats (4.41 ± 0.38 vs. 2.12 ± 0.15 μg/mg dry tissue weight, respectively, and was significantly reduced in OLETF-CR rats (2.72 ± 0.16 mg/mg wet wt) compared with OLETF-AL rats (Fig. 6d).

**Fig. 7:**
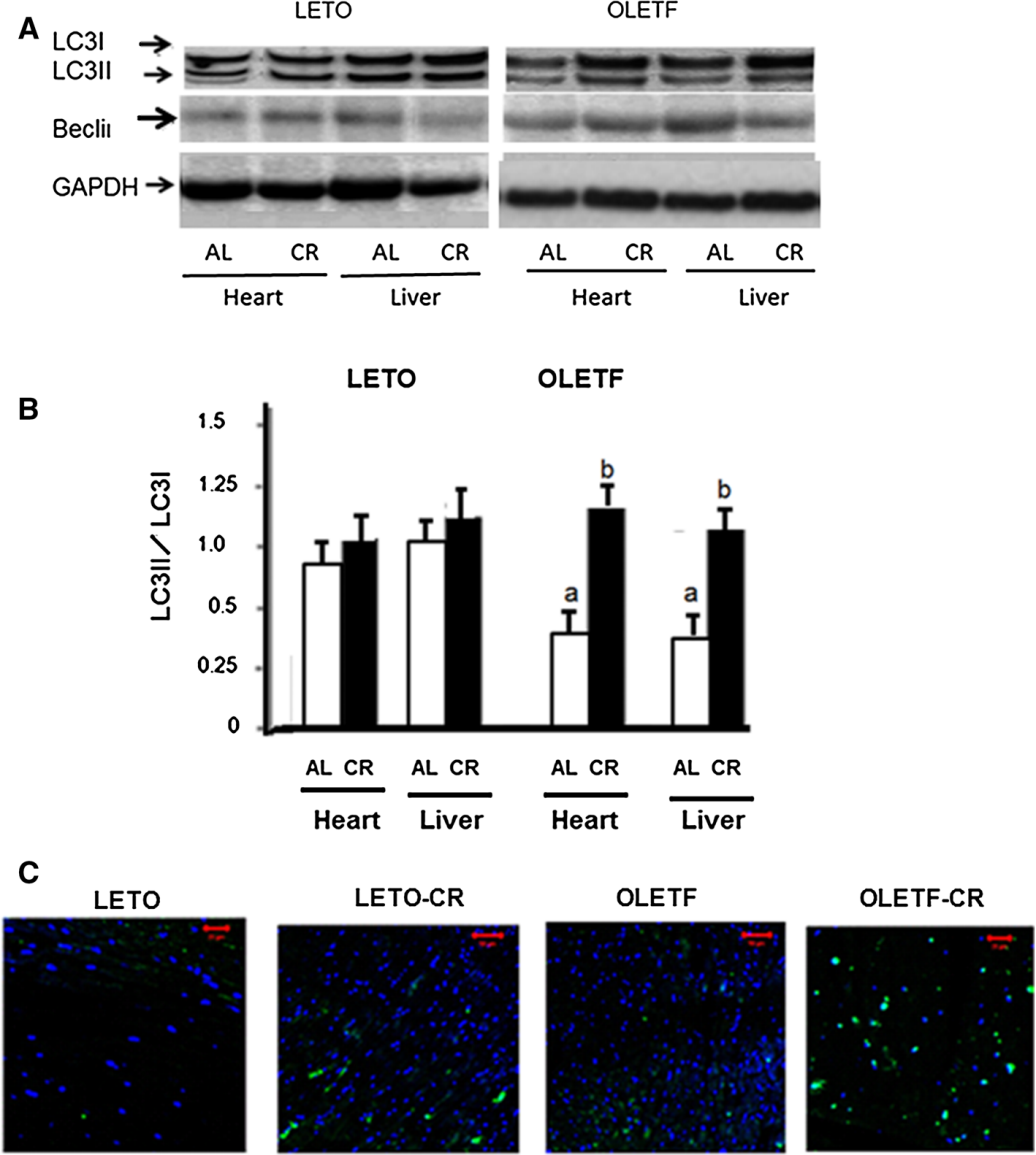
Western immunoblotting for light chain 3 (LC3) and beclin1. **a** Representative Western immunoblots showing the expression of conjugated (LC3-II), cytosolic (LC3-I) LC3, beclin1 and GAPDH. **b** Densitometric analysis of beclin1 and the LC3-II/LC3-I ratio. Data are the mean ± SE of 5 experiments. *a* *p* < 0.05 versus LETO-AL rats; *b* *p* < 0.05 versus OLETF-AL rats. **c** Immunofluorescent study of LC3 protein levels in the heart sections with green signals indicating LC3 deposition. Original magnification, ×630. DAPI staining was performed as counterstaining

Our results strongly suggest that CR has the potential to improve diastolic dysfunction in the diabetic myocardium, and that these effects might be attributable to the up-regulation of telomerase activity in addition to ameliorating oxidative DNA damage. To clarify which mechanism is mainly responsible for the intracellular signaling for cell survival, we examined the effects of CR on autophagic flux by assessing the expression of LC3-II both in experimental rats fed an AL diet and those fed a CR diet (Fig. 7a, b). An increase in the ratio of LC3-II to the cytosolic form of LC3 (LC3-I) was observed in OLETF-CR rats compared with OLETF-AL rats, but was not in LETO-CR rats. An immunofluorescent study of the levels of LC3 protein showed that the green signals indicating LC3 deposition were increased in the heart tissue of OLETF-CR rats compared to OLETF-AL rats, although those were not between LETO-AL and LETO-CR (Fig. 7c). The expression levels of beclin 1 were not significantly different among the groups in the present study.

## Discussion

This study revealed that CR increases telomerase activity, improves diastolic dysfunction, and affects cell signals for senescence, such as p-Akt and FoxO signalings, in the hearts of OLETF rats. These changes may have contributed to the weight loss and attenuation of insulin resistance in OLETF rats. CR was introduced by a 30 % reduction of daily energy intake before physical maturation in experimental rats which is required to maintain normal body weight and adiposity and is minimized to low normal levels while adequate intakes of protein and micronutrients are maintained at sufficient levels to avoid malnutrition. As Weiss EP et al. recently described [6], CR has recently been used in animal models to alter the severity of pathological/abnormal conditions and is intended to change a “normal” physiological state into a “supernormal” state in which the physiological functioning far exceeds the level required under normal conditions. The model of CR used in the present study resulted in growth retardation, and neither weight loss nor an attenuation of insulin resistance occurred, which agreed with the health and longevity effects of CR described by others [3, 6]. Thus, since many advances in understanding the effects of CR have been reported [3, 4, 17], our results may not be attributable only to weight loss. However, it is still difficult to differentiate the mechanisms between the effects of weight loss and CR.

The OLETF rats used in the present study are a hyperphagic rodent model that develop obesity, insulin resistance, and type 2 diabetes (25). Previously, we reported that there was no TL shortening observed in heart tissues in OLETF rats compared with LETO rats, whereas telomerase activity was significantly decreased in OLETF rats (10). Those results were also confirmed in the present study. It appears that the rate of TL shortening is not consistent across organs in aged animals, as TL in somatic cells reflects replicative history and predicts remaining proliferative potential. Thus, although the rate of TL shortening in each organ may be age dependent, cardiomyocytes and skeletal muscle cells are not originally proliferative and are terminally differentiated. Although previous reports have suggested that the TL attrition may be a marker associated with the presence and the number of diabetic complications [8, 9], their findings were obtained using leukocytes, not heart tissues. The present study suggested that CR may have many advantages for TL biology, diastolic dysfunction, and cardiac senescence [4]. These cardiac functional data may be associated with normalization of multiple biomarkers, including telomerase activity, which was minimal or completely recovered in OLETF rats under CR in the present experiments. Cardiac telomerase activity is detectable at the earliest stages of life and is down-regulated in the adult rat myocardium [10]. Our results on the TL were consistent with a previous study using heart tissue of the same rat models, which reported that telomerase activity was lower in OLETF rats than LETO rats [12], but here this activity was enhanced in the heart tissue of OLETF through CR. Telomere consists of tandem repeats at chromosome ends and is maintained by the catalytic subunit TERT and bound by specific TRFs, including TRF1 and TRF2 [18]. Its function is one of the principal factors in the maintenance of telomere integrity. Our results may contribute to an improved non-telomeric affect of TERT and specific TRFs without TL attrition through CR [20, 21]. It has been shown that cellular senescence can be induced without telomere shortening in dividing cells under high stress conditions [20]. On the other hand, cytosolic TERT has been shown to signal the induction of senescence by a mechanism independent of TL [21]. Thus, in terms of a mechanism by which CR could enhance telomerase activity in the diabetic heart, telomere-associated proteins are also thought to be important for the regulation of cardiac muscle cell growth and survival.

The OLETF rats used in this study are a hyperphagic rodent model that develops obesity and insulin resistance and is similar to rodent models of type II diabetes [4, 12]. In the protocol of this study, CR was undertaken as a 30 % calorie restriction for 32 weeks. Although long-term CR has been shown to reduce risk factors for cardiovascular disease [6, 17], recent evidence has also shown the cardioprotective effects of short-term CR, which involves caloric reductions similar to those in long-term CR, but for only 4–6 weeks [22, 23]. In fact, short-term CR can reduce hypertension and prevent the cardiac hypertrophy inherent to nonobese spontaneously hypertensive rats [22]. Others have described the amelioration in intracellular Ca^2+^ handling in which CR attenuated the decrease in sarcoplasmic reticulum calcium ATPase 2 protein with aging [23]. It is therefore thought that the period of CR in the present study may have been long enough to affect the aging process and improve diastolic dysfunction in diabetic rat hearts. Furthermore, the induction of the expression of age-related genes persists as long as animals remain on CR, even after energy balance is restored [24]. The present study showed protective effects of CR on diastolic function in aging diabetic rats. Other studies have reported that CR reduced cardiac oxidative stress, as measured by 8-OHdG signals, activated the autophagy process and improved myocardial protein degradation [14, 25]. Although the mechanisms by which long-term CR retards cellular senescence and attenuates the physiological decline of organ function have not been fully elucidated, it is known that aging occurs in part as a result of the accumulation of the end products of oxidative damage caused by oxidative free radicals that are generated continuously during the course of metabolic processes [26]. CR also decreases the age-associated accumulation of the end products of oxidative damage to lipids, proteins, and DNA [17, 21]. Thus, it is possible that long-term CR retards cellular senescence and ameliorates age-related functional decline by attenuating oxidative damage in the diabetic heart. Our data suggest that long-term CR induced autophagy. In regard to autophagy during CR, it is now considered that under basal conditions autophagy can play a housekeeping role in the turnover of cytoplasmic constituents [5, 16, 19] and can confer protection by degrading and removing damaged organelles and accumulated protein aggregates. However, it is controversial whether autophagy is the cause of cell death or acts as a compensatory mechanism to protect cells from death. Prior research suggests that autophagy has two opposing effects in impaired cardiomyocytes: one is the protection of cells from death and the other is the promotion of cell death [25, 27]. Since CR induces protein expressions of Mn-SOD as well as eNOS and reduces oxidative stress in the heart [4], the effects of autophagy observed herein may function as a compensatory mechanism to protect cells from death. In fact, CR decreased the level of caspase-3 protein expression in OLETF rats, and caspase-3 protein expression is thought to promote apoptosis in heart tissues.

In conclusion, the present study provides the first evidence of the beneficial effects of CR namely; CR was shown to increase telomerase activity and to improve diastolic dysfunction in the hearts of diabetic rats. These results may have been partly attributable to enhanced autophagy flux, and attenuated muscle protein degradation through CR. In addition, CR delayed the progression of diastolic dysfunction in diabetic hearts of OLETF rats. Together, our data suggest that CR could be of benefit in patients with diabetes, and may activate a metabolic switch that translates the dietary changes into a program of health and survival. However, despite the positive benefits of CR reported herein, compliance remains a major impediment, because the lifestyle changes associated with CR require considerable patient commitment. Elucidating the major mechanism underlying the improvement of heart function through CR will require much more investigation.

## References

[1] Mizushige K, Yao L, Noma T, Kiyomoto H, Yu Y, Hosomi N, Ohmori K, Matsuo H (2000). Alteration in left ventricular diastolic filling and accumulation of myocardial collagen at insulin-resistant prediabetic stage of a type II diabetic rat model. Circulation.

[2] Goraya TY, Leibson CL, Palumbo PJ, Weston SA, Killian JM, Pfeifer EA, Jacobsen SJ, Frye RL, Roger VL (2002). Coronary atherosclerosis in diabetes mellitus: a population- based autopsy study. J Am Coll Cardiol.

[3] Mehta LH, Roth GS (2009). Caloric restriction and longevity: the science and the ascetic experience. Ann N Y Acad Sci.

[4] Minamiyama Y, Bito Y, Takemura S, Takahashi Y, Kodai S, Mizuguchi S, Nishikawa Y, Suehiro S, Okada S (2007). Calorie restriction improves cardiovascular risk factors via reduction of mitochondrial reactive oxygen species in type II diabetic rats. J Pharmacol Exp Ther.

[5] Shinmura K, Tamaki K, Sano M, Murata M, Yamakawa H, Ishida H (2011). Impact of long-term caloric restriction on cardiac senescence: caloric restriction ameliorates cardiac diastolic dysfunction associated with aging. J Mol Cell Cardiol.

[6] Weiss EP, Fontana L (2011). Caloric restriction: powerful protection for the aging heart and vasculature. Am J Physiol Heart Circ Physiol.

[7] Adaikalakoteswari A, Balasubramanyam M, Mohan V (2005). Telomere shortening occurs in Asian Indian Type 2 diabetic patients. Diabet Med.

[8] Gardner JP, Li S, Srinivasan SR, Chen W, Kimura M, Lu X, Berenson GS, Aviv A (2005). Rise in insulin resistance is associated with escalated telomere attrition. Circulation..

[9] Testa R, Olivieri F, Sirolla C, Spazzafumo L, Rippo MR, Marra M, Bonfigli AR, Ceriello A, Antonicelli R, Franceschi C, Castellucci C, Testa I, Procopio AD (2011). Genetics leukocyte telomere length is associated with complications of Type 2 diabetes mellitus. Diabet Med.

[10] Borges A, Liew CC (1997). Telomerase activity during cardiac development. J Mol Cell Cardiol.

[11] Wong LS, Oeseburg H, de Boer RA, van Gilst WH, van Veldhuisen DJ, van der Harst P (2009). Telomere biology in cardiovascular disease: the TERC^−/−^ mouse as a model for heart failure and ageing. Cardiovasc Res.

[12] Makino N, Maeda T, Oyama J, Higuchi Y, Mimori K (2009). Improving insulin sensitivity via activation of PPAR-γ increases telomerase activity in the heart of OLETF rats. Am J Physiol Heart Circ Physiol.

[13] Herbig U, Ferreira M, Condel L, Carey D, Sedivy JM (2006). Cellular senescence in aging primates. Science.

[14] Cuervo A (2004). Autophagy in sickness and in health. Trends Cell Biol.

[15] Makino N, Maeda T, Oyama J, Sasaki M, Higuchi Y, Mimori K, Shimizu T (2011). Antioxidant therapy attenuates myocardial telomerase activity reduction in superoxide dismutase-deficient mice. J Mol Cell Cardiol.

[16] Mizushima N, Yoshimori T (2007). How to interpret LC3 immunoblotting. Autophagy.

[17] Sinclair DA (2008). Toward a unified theory of caloric restriction and longevity regulation. Mech Ageing Dev.

[18] Cohen SB, Graham ME, Lovrecz GO, Bache N, Robinson PJ, Reddel RR (2007). Protein composition of catalytically active human telomerase from immortal cells. Science.

[19] Iwai-Kanai E, Yuan H, Huang C, Sayen MR, Perry-Garza CN, Kim L, Gottlieb RA (2008). A method to measure cardiac autophagic flux in vivo. Autophagy.

[20] Farhat N, Thorin-Trescases N, Voghel G, Villeneuve L, Mamarbachi M, Perrault LP, Carrier M, Thorin E (2008). Stress-induced senescence predominates in endothelial cells isolated from atherosclerotic chronic smokers. Can J Physiol Pharmacol.

[21] Haendeler J, Hoffmann J, Diehl JF, Vasa M, Spyridopoulos I, Zeiher AM, Dimmeler S (2004). Antioxidants inhibit nuclear export of telomerase reverse transcriptase and delay replicative senescence of endothelial cells. Circ Res.

[22] Dolinsky VW, Morton JS, Oka T, Robillard-Frayne I, Bagdan M, Lopaschuk GD, Des Rosiers C, Walsh K, Davidge ST, Dyck JR (2010). Calorie restriction prevents hypertension and cardiac hypertrophy in the spontaneously hypertensive rat. Hypertension.

[23] Kondo M, Shibata R, Miura R, Shimano M, Kondo K, Li P, Ohashi T, Kihara S, Maeda N, Walsh K, Ouchi N, Murohara T (2009). Caloric restriction stimulates revascularization in response to ischemia via adiponectinmediated activation of eNOS. J Biol Chem.

[24] Spindler SR, Dhahbi JM (2007). Conserved and tissue-specific genic and physiologic responses to caloric restriction and altered IGF1 signaling in mitotic and postmitotic tissues. Annu Rev Nutr.

[25] Cuervo AM, Bergamini E, Brunk UT, Droge W, French M, Terman A (2005). Autophagy and aging: the importance of maintaining “clean” cells. Autophagy.

[26] Sukhanov S, Higashi Y, Shai SY, Vaughn C, Mohler J, Li Y, Song YH (2007). IGF-1 reduces inflammatory responses, suppresses oxidative stress, and decreases atherosclerosis progression in ApoE-deficient mice. Arterioscler Thromb Vasc Biol.

[27] Baehrecke EH (2005). Autophagy: dual roles in life and death?. Nat Rev Mol Cell Biol.

